# Conditions for Shake-Gel Formation: The Relationship between the Size of Poly(Ethylene Oxide) and the Distance between Silica Particles

**DOI:** 10.3390/molecules27227770

**Published:** 2022-11-11

**Authors:** Yi Huang, Shunsuke Sato, Motoyoshi Kobayashi

**Affiliations:** 1 Graduate School of Life and Environmental Sciences, University of Tsukuba, 1-1-1 Tennoudai, Tsukuba 305-8572, Japan; 2 Graduate School of Science and Technology, University of Tsukuba, 1-1-1 Tennoudai, Tsukuba 305-8572, Japan; 3 Faculty of Life and Environmental Sciences, University of Tsukuba, 1-1-1 Tennoudai, Tsukuba 305-8572, Japan

**Keywords:** bridging effect, radius of gyration, molecular weight, distance between silica particles, Woodcock’s equation

## Abstract

Colloidal silica suspensions are widely used in many fields, including environmental restoration, oil drilling, and food and medical industries. To control the rheological property of suspensions, poly(ethylene oxide) (PEO) polymers are often used. Under specific conditions, the silica-PEO suspension can create a phenomenon called a shake-gel. Previous works discussed the conditions necessary to form a shake-gel and suggested that the bridging effect of the polymer is one of the important mechanisms for shake-gel formation. However, we noted that the influence of PEO size compared to the separation distance between silica particles regarding shake-gel formation has not been systematically investigated, while the PEO size should be larger than the particle–particle separation distance for polymer bridging in order to form gels. Thus, we conducted a series of experiments to examine the effects of the radius of gyration of the PEO and the distance between the silica particles by controlling the PEO molecular weight and the silica concentration. Our results elucidated that the radius of gyration of the PEO should be 2.5 times larger than the distance between the silica surfaces in order to promote the formation of a shake-gel. This result supports the hypothesis that the bridging effect is the main cause of shake-gel formation, which can help us to understand the conditions necessary for shake-gel preparation.

## 1. Introduction

Aqueous colloidal suspensions are encountered in many fields. Specifically, aqueous colloidal silica suspensions are widely used in environmental restoration [1], oil drilling, food [2], and medical industries [3]. In these areas, controlling the state and the rheological properties of silica suspensions is important. For this end, polymers are often added to silica suspensions. The added polymers adsorb to the surface of silica particles, and the characteristics of the silica surfaces change. Due to this adsorption behavior, polymers alter the interaction between silica particles, and this alteration also induces the various states of the suspension [4,5]. Therefore, it is important to understand the role of polymers in affecting the rheological properties of colloidal suspensions.

Many colloidal suspensions, with or without polymers, change their viscosity with a change in shear rate [6,7,8,9]. One phenomenon occurring due to an increase in viscosity with shear rate is called shear-thickening. Along with the increase in viscosity, some of the suspensions show gel-like behaviors with shaking and shear flow. These gels are called shake-gels [9,10,11]. The shake-gel can even show the phenomenon of relaxation; that is, shake-gels in a gel state can turn back to a sol state after being left standing. Because of this interesting characteristic, several previous studies were conducted to reveal the mechanism of shake-gel formation. Cabane et al. [9] conducted experiments on shake-gels made up of suspensions with silica particles and poly(ethylene oxide) (PEO) and reported that the mixed suspensions can become shake-gels. They observed the states, gelled or not, of silica-PEO suspensions under several silica and PEO concentrations. Their results confirmed that the suspensions can become shake-gels at appropriate silica and PEO concentrations. Zebrowski et al. [10] reported that the mixed suspensions of synthetic clay particles laponite and PEO showed the shake-gel phenomenon. Their study demonstrated that the shake-gel phenomenon occurred in a limited range of laponite and PEO concentrations, which is similar to the results of Cabane et al. [9]. Mar Ramos-Tejada et al. [11] also prepared shake-gels with silica, laponite, montmorillonite particles, and PEO polymers to examine the states of the suspensions under different weight fractions of nanoparticles and PEO. Their results were summarized into state diagrams by using the PEO dose per particle surface area (shown as *C*_p_ in this paper) to evaluate the PEO coverage instead of PEO concentration. As a result, the diagrams showed that the suspensions can become shake-gels if the *C*_p_ is in an appropriate range, even though the particle concentration is different. This conclusion is important and indicates that PEO coverage on particles, rather than concentration, is the definitive factor in the formation of shake-gels.

The previous studies mentioned above used different materials and methods to observe the state and rheological properties of shake-gels. From their results, they hypothesized that the bridging effect of PEOs is a prerequisite for forming shake-gels. In this hypothesis, added each PEO chain can adsorb to several particles and form suspended small PEO-silica aggregates. Before shaking, PEOs maintain a random-coil-like state, and thus the suspension behaves as a sol. When the suspension is shaken, shear flow stretches the PEOs or PEO-silica clusters into an elongated state. Elongated PEOs or clusters build more bridges between the small aggregates, inducing the formation of a reversible gel network. The gel network is unstable because the PEOs and clusters tend to return to the suspended random-coil-like and undeformed state due to thermal motion, as well as electric double layer repulsion between silica particles, once the shear flow is stopped. Therefore, after stopping the shaking, the network breaks up over time due to the bridging, and the shake-gels relax to a sol state. Saito et al. [12], Shibayama et al. [13], and Tian et al. [14] also illustrated similar physical depictions of the gelation and relaxation process.

Focusing on this hypothesis, we have noticed that parameters influencing the bridging of PEO between colloidal particles may also affect the formation of shake-gels. Possible parameters affecting the formation of shake-gels are silica and PEO concentrations, pH, molecular weight of the PEO, and temperature. The studies mentioned above reported that higher particle concentrations promoted the formation of a shake-gel. The effect of pH on shake-gels was first examined by Kawasaki et al. [15], who showed that shake-gels only occurred in the pH range of 8.0–9.9 in silica-PEO suspensions. A previous work [16] also revealed the effect of pH and PEO molecular weight on the formation of shake-gels, probably because pH influences the electrical double layer repulsion between silica particles [17,18,19], and PEO molecular weight affects the number of crosslinks of PEO and silica [11]. The longest relaxation time was found around *C*_p_~0.04 mg∙m^−2^ [16]. Pozzo et al. [20] and Collini et al. [21] indicated that the temperature of the system is also an important parameter involved in the relaxation and gelation time of the shake-gels. In the gelation and relaxation of the shake-gels, the temperature is related to the kinetic energy and migration velocity of particles in the system. From these works, it seems that the possible parameters related to the shake-gel were implicitly identified.

However, we recently realized that the parameters mentioned above have so far been discussed separately. The simultaneous combination of such parameters into an integrated hypothesis can help us to find the conditions intrinsic to the formation of shake-gels. In this study, we have examined the effect of particle concentration and PEO molecular weight, mainly around optimum *C*_p_. Particle concentration is related to the distance between particles, and the molecular weight of PEO restricts the maximum length of the polymer bridges. Yamagata et al. [22] devised a similar hypothesis and found that the shake-gel of the bentonite/heptaethylene oleyl ether system only occurs when the concentration of bentonite is greater than 1.3 wt% [22]. It is highly possible that a specific relationship between the particle concentration (particle–particle distance) and the molecular weight of PEO (PEO size) exists for the appearance of shake-gels.

Hence, we devised a hypothesis that, in addition to *C*_p_, another condition necessary to form a shake-gel is that the PEO polymer should be long enough to build bridges between the colloidal particles. We studied shake-gels containing silica nanoparticles and PEO under different *C*_p_, PEO molecular weights, and silica concentrations to verify our hypothesis. We focused on the average distance between silica particles and the *R*_g_ of PEO polymers in this research, and found that the temperature does not significantly affect the average distance of silica and the *R*_g_ of PEO polymers at room temperature. Therefore, we conducted our experiment only at the room temperature (20 °C). This study is valuable to deepen our understanding of the mechanism of shake-gel formation.

## 2. Materials and Methods

### 2.1. Materials

Silica nanoparticle suspension (LUDOX TM-50, Sigma-Aldrich, Tokyo, Japan) was used as colloidal suspension in this study. We used the silica as received, without any further purification. The mass fraction of stock silica suspension is 49.9%. The hydrodynamic diameter was measured to be about 32.35 ± 0.22 nm [15], and the specific surface area *S*_p_ was about 140 m^2^/g, according to the manufacturer. With the values of *S*_p_, the added mass of PEO *m*_p_, and the silica mass *m*_s_, the PEO dose per particle surface area of *C*_p_ can be calculated using *C*_p_ = *m*_p_/(*S*_p_*m*_p_), with proper unit conversion.

Poly(ethylene oxide) (PEO) is a non-ionic linear polymer. PEO powders were purchased from Sigma-Aldrich and used without any purification. The average PEO molecular weights we used were 400, 600, 1000, 2000, and 4000 kDa (product ID: 372773-250G, 182028-250G, 372781-250G, 372803-250G, and 189464-250G, respectively). The PEO powder was dissolved in deionized water to prepare stock solutions. Stock PEO solutions were stirred for about 72 h under darkness to ensure complete dissolution. Szekely et al. [23] reported that polymers synthesized by the classical polymerization are polydisperse. We thus consider that the polymers we used are polydisperse, without requiring more details about the synthesis methods from the manufacturer. Deionized water (Elix Advantage 5, Millipore, Tokyo, Japan) was used to prepare the solutions and suspensions in this study. The electric conductivity of freshly prepared deionized water was about 0.07 μS/cm.

### 2.2. Methods

Silica-PEO suspensions were prepared in glass test tubes. Silica suspension and PEO stock solution described above were used to prepare the silica-PEO suspensions. The total mass of the silica-PEO suspensions was set to 2 g. The mass fraction of the silica particles was changed from 5 to 30% by an increase of 5%. The dose of PEO solution was changed to vary the added mass of PEO per unit silica surface area (*C*_p_) from 0.02 to 0.15 mg∙m^−2^. While the adsorbed amount of PEO may be a better indicator in this study, the adsorbed amount may change during gelation and relaxation processes. Therefore, we use *C*_p_ instead of the adsorbed amount. The additive amounts of these materials were calculated beforehand, and we added them in the order of silica suspension, deionized water, and the PEO solution in the test tube. Prepared silica-PEO suspensions were pre-mixed using a test tube mixer (PresentMixer, TAITEC, Koshigaya, Japan) for several minutes. In the pre-mixing process, some suspensions gelled. To ensure the complete relaxation of the suspensions to sol, the suspensions were left to stand for about 24 h.

To shake the suspensions, we used the same test tube mixer as used in the pre-mixing process. The mixer can provide rotations to the test tubes, and the rotation speed is about 2800 rpm. When 24 h had passed for relaxation after the pre-mixing, we pressed the test tubes to the mixer lightly and vertically. The PEO-silica suspensions require a certain period of mixing time to gel [21]. In our previous study, we found that almost all the suspensions gelled in about 10 s of the mixing using the test tube mixer [16]. Therefore, to ensure the gelation of all the suspensions, we shook the samples for 60 s. Immediately after the shaking process, we stopped the mixer and took photos to observe the state of the suspensions. The mixed suspensions of silica and PEO showed several states after the shaking. Their states have been differently defined by many studies [9,11,15,16]. We continue to use our classification of states [16]. Immediately after the shaking, the test tube was laid down. Then, if the suspension slid or flowed down from the upper wall of the test tube, we judged it was in a sol state. If not, we defined it as a gel state. By this method, the silica-PEO suspensions can be classified into the states of cloudy, permanent gel, shake-gel, or high viscosity sol according the classifications determined in our previous publication [16]. From this observation, we made diagrams of states of silica-PEO suspensions and clarified the conditions necessary to form a shake-gel. After taking photos, we measured the pH of the suspensions using a compact pH meter (LAQUA twin pH-22B, HORIBA, Kyoto, Japan).

In addition, we performed viscosity measurements of the silica-PEO suspensions at several selected conditions to confirm the state diagram based on direct observation. The suspensions in the relaxed sol-like state were applied to shear flow by using a concentric double-cylinders rheometer (Merlin VR, Rheosys, Princeton, NJ, USA). The viscosity of the samples was measured using varying shear rates. The sol state samples were placed in the rheometer and left to settle for 15 min before the measurements began to offset the history of shear applied to the samples when they were set in the rheometer. The shear rate changed from 10 to 2000 s^−1^ in 30 steps, and each measurement was performed for 30 s. The analysis was performed from 10 to 1388 s^−1^ because samples with low viscosity overflowed from the measurement system at shear rates higher than 1666 s^−1^.

## 3. Results and Discussion

### 3.1. Some Notes from Previous Findings on the Shake-Gel Formation of Silica-PEO Suspensions

In this sub-section, before showing the main results of the present study, we briefly introduce previously reported related findings [16]. We found that shake-gel can be obtained at appropriate ranges of *C*_p_, and pH was also an important factor for the state. The present study focuses on the results at a pH of about 9.4 because silica-PEO suspensions more easily attained the shake-gel phenomenon and could also relax to sol within a reasonable time at this pH. Furthermore, our silica-PEO suspensions showed a pH of around 9.4 without any pH adjustment.

For the samples of shake-gels, we have noted the difference in the time required to return to the sol state. We have called the time to return to sol a relaxation time. The relaxation time was quantified from the direct observation of relaxing shake-gel for samples with different molecular weights of PEO [16]. This relaxation time was sensitive to the change in *C*_p_ and the molecular weight of PEO. All silica-PEO suspensions had the maximum relaxation time of around *C*_p_ = 0.04–0.05 mg∙m^−2^ [16]. We consider that an appropriate coverage of PEOs on the silica surface is necessary for the formation of a shake-gel. When the *C*_p_ was in the range of 0.04–0.05 mg∙m^−2^, the shake-gels were relatively stable, maintaining the gel state for longer time that that noted in the data of ref. [16]. We also found that the maximum relaxation time increased with the molecular weight of the PEO. Thus, it should be noted that the molecular weight of the PEO and the choice of *C*_p_ can be important parameters to discuss regarding the formation and properties of a shake-gel.

### 3.2. State Diagram of Silica-PEO Suspensions Consisting of Different PEO Molecular Weights

**Figure 1:**
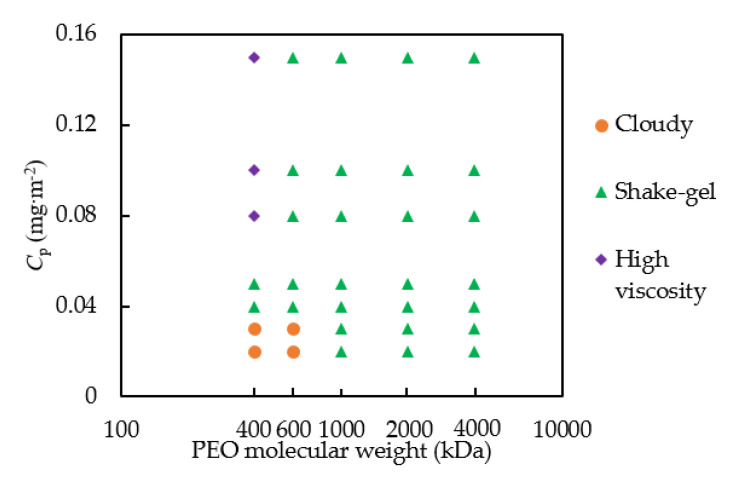
The state diagram of the silica-PEO suspension. The silica concentration was fixed to 20 wt%; the PEO molecular weight was changed to 400, 600, 1000, 2000, and 4000 kDa; and the pH was 9.4. *C*_p_ is the PEO dose per silica surface area.

The states diagram of the silica-PEO suspensions under different *C*_p_ and PEO molecular weights at the weight fraction for silica of 20 wt% is shown in Figure 1. The different symbols represent different states of the silica-PEO suspensions. We can confirm the effect of molecular weights of PEO polymers on the state change of silica-PEO suspensions. 

For these PEO polymers with different molecular weights, all the suspensions can form shake-gels under specific conditions. However, we found that suspensions consisting of 400 kDa PEO can produce cloudy, shake-gel, and high viscosity sol states. Meanwhile, suspensions consisting of 600 kDa PEO have a larger *C*_p_ area of shake-gel than that of the 400 kDa case, and the high viscosity sol state disappears. When the molecular weights of PEO were higher than 1000 kDa, the suspensions can form shake-gels under all the examined conditions.

We consider that the above state results are due to the size of the PEO. As a reference for PEO size, the radius of gyration *R*_g_ of PEO can be calculated by

(1) $$R_{g}=\sqrt{4.08\times 10^{-4}\times M_{w}^{1.16}}$$

where *M*_w_ is the molecular weight of the PEO [15]. The radius of gyration *R*_g_ is 36 nm for the 400 kDa PEO polymer. This smaller size of 400 kDa PEOs makes it difficult to bind multiple silica particles using one polymer. Furthermore, shorter PEOs show difficulty in forming a network structure, instead spreading throughout the whole suspension. Therefore, the particles aggregate by bridging to larger flocs. These flocs strongly scatter light and make the suspensions cloudy. For PEO polymers with 600, 1000, 2000, and 4000 kDa, the values of *R*_g_ are about 46, 61, 91, and 136 nm, respectively [24,25]. These polymer sizes are larger than that of the 400 kDa PEO polymers and can be stretched to be longer by shear force. Therefore, the use of one PEO chain can more easily bind additional silica particles, and thus high molecular weight polymers promote the formation and prevent the relaxation of gels.

### 3.3. State Diagram of Silica-PEO Suspensions under Different Silica Concentrations

**Figure 2:**
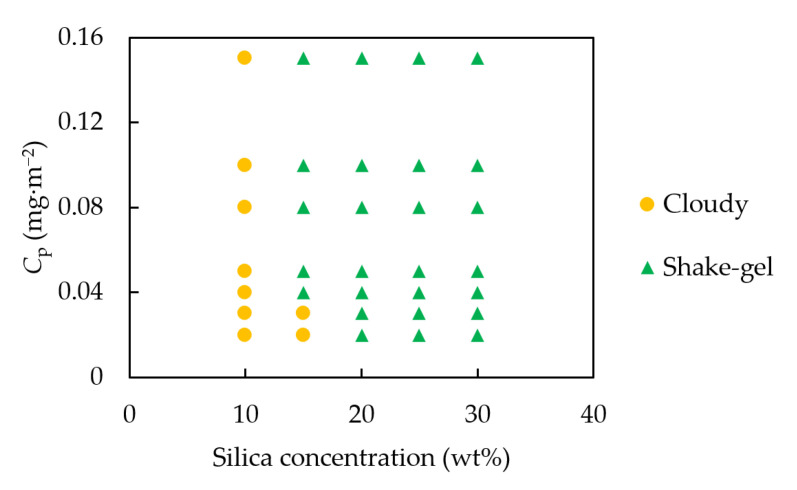
The state diagram of the silica-PEO suspension. The silica concentration was set to 10, 15, 20, 25, and 30 wt%. The PEO molecular weight was 1000 kDa; the pH was 9.4. *C*_p_ is the dose of PEO per unit of the silica surface area.

As discussed above, we devised a hypothesis that one PEO polymer must bind multiple silica particles to form shake-gels. This hypothesis simply suggests that not only the size of the PEO, but also the distance between silica particles, can be a definitive parameter to affect the formation of a shake-gel. Moreover, in the range of PEO concentrations in this research, we did not observe the gelation of pure PEO solutions. Therefore, the existence of silica particles and the distance between them should play an important role in the gelation of silica-PEO suspensions, perhaps by acting as the crosslinkers between PEO polymers [9,26]. To verify our hypothesis, we changed the silica concentration to control the distance between silica particles and observed the state of the silica-PEO suspension. The results are shown in Figure 2.

In Figure 2, the data using only the PEO polymer of 1000 kDa are plotted. When the silica concentration decreased to 10 wt%, all suspensions showed the cloudy sol state and the shake-gel did not appear. For suspensions of 15 wt% silica, part of the suspensions turned to gel. By further, increasing the silica concentration to 20, 25, and 30 wt%, we observed the shake-gel formation in all the examined suspensions. Consequently, it is now clear that higher silica concentrations are necessary for the formation of shake-gels.

The reason why higher silica concentrations promoted gelation is the existence of shorter distances between the particles and an increase in the crosslinkers between the PEOs. As reported in Gaharwar et al. [26], silica particles can be the crosslinkers of PEOs. Due to the existence of crosslinkers, PEOs can build networks. When the silica concentration is low, the number of concentration of crosslinkers is also low. Therefore, PEOs entangle silica particles to make flocs, and suspensions show only a cloudy state. If the silica concentration increases, the crosslinkers also increase. This makes it easier for the PEOs to form a gel network in the suspension. Thus, we can confirm the shake-gel state in a wider *C*_p_ range at higher silica concentrations.

### 3.4. The Gelation of a Silica-PEO Suspension Due to the Relationship between PEO Molecular Weight and Silica Concentration

Through the experimental results shown above, we found that larger PEO polymers and higher silica concentrations promote the formation of shake-gels. This trend can be explained by the fact that larger PEO polymers are easier to bind to silica particles. Conversely, these results also mean that it is impossible to form shake-gels in silica-PEO suspensions if the PEO polymers are too small or the distance between silica particles is too large. Therefore, we discuss the relationship between the *R*_g_ of PEO polymers and the average distance of the surfaces between the nearest silica particles.

The *R*_g_ of PEO can be calculated by Equation (1) and is determined only by the molecular weight. As for the average distance between the silica particles, we assume silica particles are distributed in the suspension with a hexagonal close-packed structure. With this assumption, we can calculate the average surface distance of the nearest silica particles *h* using Woodcock’s equation [27], that is,

(2) $$h=d\left(\sqrt{\frac{1}{3\pi \phi }+\frac{5}{6}}-1\right)$$

where *d* and *ϕ* are the diameter and the volume fraction of silica particles, respectively.

**Figure 3:**
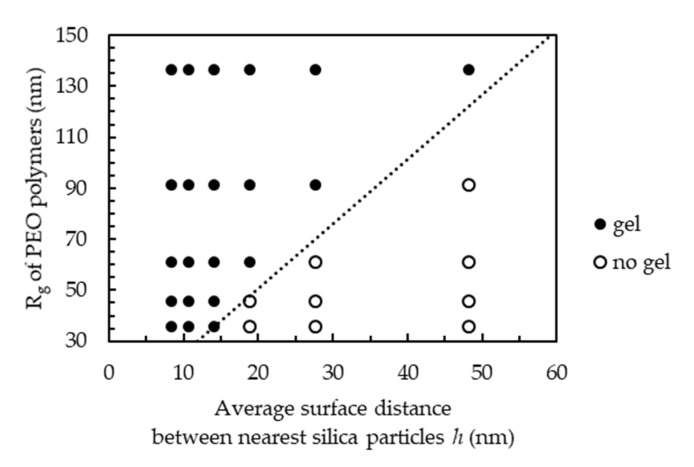
The state diagram of silica-PEO suspensions with various molecular weight PEOs and concentrations of silica. *C*_p_ was fixed to 0.04 mg∙m^−2^. The average distance of silica and the *R*_g_ of PEO were calculated according to the silica concentration (5, 10, 15, 20, 25, 30 wt%) and the PEO molecular weight. The borderline is around *R*_g_ = 2.5 *h*, indicating that the cause of gelation is the bridging effect.

The states diagram in terms of *R*_g_ and *h* is shown in Figure 3, where the experimental data were taken at *C*_p_ = 0.04 mg∙m^−2^ because stable shake-gels can more easily form at this *C*_p_, as described above. We can confirm that both the increase in *R*_g_ of the polymers and the decrease in *h* can promote the gelation of silica-PEO suspensions. The borderline between gel and non-gel states seems to be a linear line in which the *R*_g_ is about 2.5 times the distance of *h*. Our previous results demonstrated that *C*_p_= 0.04 mg/m^2^ is the best *C*_p_ condition for shake-gel formation at the fixed conditions of pH, silica concentration, and PEO molecular weight. Even under the best *C*_p_ condition, shake-gels do not form when *R*_g_ is below 2.5 *h*.

**Figure 4:**
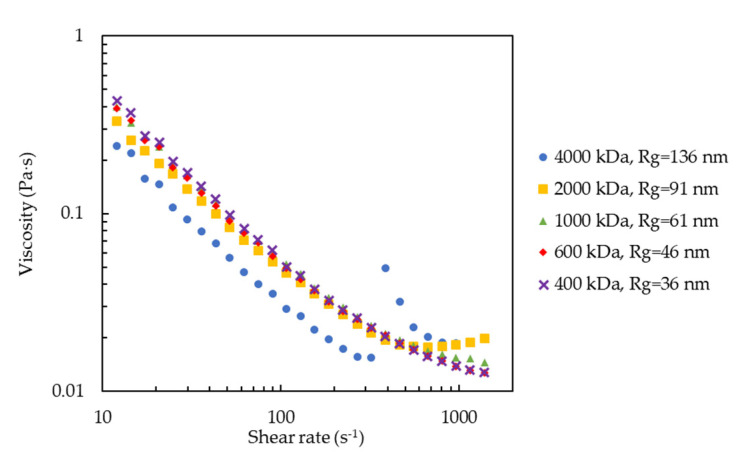
The viscosity of silica-PEO suspensions against shear rate at *C*_p_ = 0.04 mg∙m^−2^. The silica concentration was fixed to 10 wt% (*h* = 28 nm). The different symbols stand for different PEO molecular weights. In this condition, only the suspension with 2000 kDa and 4000 kDa PEO showed shear-thickening, meaning that they gelled. These results agreed with those in Figure 3, showing that the suspensions with *R*_g_ above 91 nm gelled at *h* = 28 nm.

**Figure 5:**
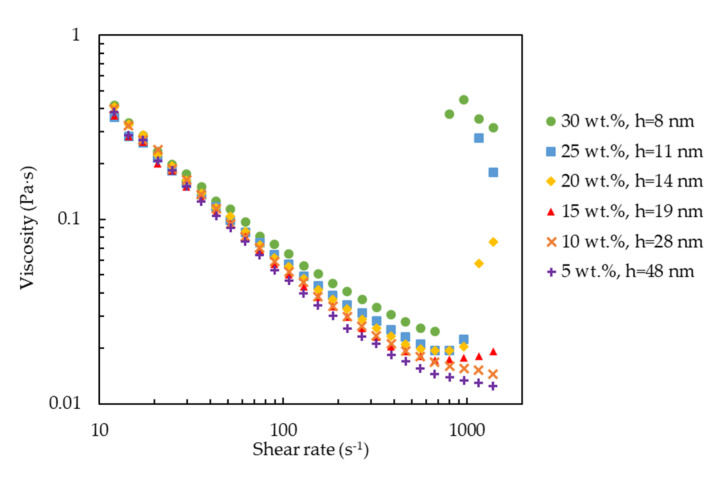
The viscosity of silica-PEO suspensions against shear rate at *C*_p_ = 0.04 mg∙m^−2^. The PEO molecular weight was fixed to 1000 kDa (*R*_g_ = 61 nm). The different symbols denote different silica concentrations. In this condition, when the silica concentration was above 15 wt. %, the suspensions showed shear-thickening, meaning that they gelled. These results agreed with those in Figure 3 showing that the suspensions with *h* below 19 nm gelled at *R*_g_ = 61 nm.

We also measured the viscosity of the silica-PEO suspensions against the shear rate to confirm if our visual observations were correct. The results of a silica concentration of 10 wt% and a PEO molecular weight of 1000 kDa, corresponding to the results of *h* = 28 nm and *R*_g_ = 61 nm, are shown in Figure 4 and Figure 5, respectively. Both the figures demonstrate that the silica-PEO suspensions show shear-thinning at lower shear rates. Shear-thickening is demonstrated for 2000 kDa (*R*_g_ = 91 nm) and 4000 kDa (*R*_g_ = 136 nm) at 10 wt% silica (*h* = 28 nm) and 15, 20, 25, and 30 wt% silica (*h* = 19, 14, 11, and 8 nm) with the molecular weight of 1000 kDa (*R*_g_ = 61 nm). The shear-thickening indicates the formation of a shear-induced gel or shake-gel. Therefore, we confirm that the results of the viscosity measurements show good agreement with the results of our visual observations.

As reported by previous studies [9,10,11,12,13], the bridging effect is the possible main cause of the gelation of the silica-PEO suspension. Therefore, whether the PEOs are long enough to build bridges between the surfaces of the silica particles is an important factor for gelation. If *h* is short or the *R*_g_ of the PEOs is large, the suspensions can form gels. When *h* is long and the *R*_g_ of PEOs is small, we find that the suspensions show a cloudy state. The thickness of the adsorption layer of the PEO may be smaller than the 2*R*_g_ because PEOs are soft and can change their conformation during the adsorption process [28,29]. Thus, to form the gel network among silica particles, the *R*_g_ of the PEOs should be longer than 2.5 *h*.

In this study, we examined the effect of linear PEO at a fixed temperature. The rheology of mixtures of a block copolymer with a PEO branch and nanosilica showed strong temperature dependence [30]. Thus, the influence of temperature and branched PEO and/or co-polymers on the formation of a shake-gel would be worthy of future investigation.

## 4. Conclusions

In this paper, we hypothesized that the radius of the gyration of the PEO should be longer than the average distance between the nearest silica particles in order to form shake-gels. To verify this hypothesis, we carried out the experiments of shake-gel formation while controlling the radius of the gyration of the PEO and the distance between the silica particles by changing the PEO molecular weight and the silica concentration. From the experiments, we confirmed that a high PEO molecular weight and a high silica concentration promoted the formation of a shake-gel. We also found that the silica-PEO suspensions can show exhibit a shake-gel state when the radius of the gyration of the PEO is 2.5 times longer than the surface–surface distance between the silica particles. This result supports the hypothesis that the bridging effect of PEO is the main cause of shake-gel formation, providing useful information for the preparation of shake-gels.

## Data Availability

Data are available from the authors upon reasonable request.

## Sample Availability

Materials are commercially available from the manufactures.
